# Conducting a registry-based randomised trial (REDOX) in chronic respiratory failure: experiences and advice

**DOI:** 10.1080/20018525.2025.2502237

**Published:** 2025-05-15

**Authors:** Josefin Sundh, Magnus Ekström

**Affiliations:** a Faculty of Medicine and Health, Department of Respiratory Medicine, Örebro University, Örebro, Sweden; b Faculty of Medicine, Department of Clinical Sciences Lund, Respiratory Medicine, Allergology and Palliative Medicine, Lund University, Lund, Sweden

**Keywords:** hypoxemia, oxygen therapy, hospitalisation, mortality, trial design

## Abstract

Registry-based randomised controlled trials (R-RCTs) represent a paradigm shift in research, with the potential to accomplish pragmatic but large trials with high external validity. In this paper, we review our experiences from planning and performing the *REgistry-based randomised controlled trial of treatment Duration and mortality in long-term OXygen therapy (REDOX) trial*, the first R-RCT within respiratory medicine. The REDOX study compared the two established treatment options of home oxygen 15 and 24 h per day. Previous recommendations to use oxygen for at least 15 h but preferably 24 h per day were based on a non-randomised comparison of two different studies. We hypothesised that oxygen 24 h/day was non-superior to 15 h/day and used the Swedish National Registry for Respiratory Failure (Swedevox) to perform an R-RCT showing that home oxygen 24 h/day does not improve survival, hospitalisation or patient-reported outcomes within 1 year. We describe the entire procedure of REDOX from planning to publication and use it to discuss challenges and potential solutions for future R-RCTs. In summary, common features of R-RCTs are the use of a registry for identification and randomisation of participants and for reporting and collecting baseline and outcome data, and the design is typically used to compare two treatment options. Important strengths are high generalisability, low cost, feasibility for consecutive recruitment in clinical practice, and high completeness of follow-up. Limitations include that coverage, completeness and accuracy of baseline data may differ between registries. Specific challenges (and solutions) in REDOX were addressing an important question (pragmatic clinical trials), management (clinical research support teams), costs (using registry-based infrastructure), different electronic data capture systems (posttrial linkage), slow recruitment (amendment of protocol) and resistance to challenge treatment traditions.

## Introduction

Long-term oxygen therapy (LTOT) is an established treatment to prolong survival in people with chronic severe hypoxemia, based on two trials from the 1970s, and was the first therapy that was shown to improve prognosis in chronic obstructive pulmonary disease (COPD) [1,2]. Based on the original efficacy trials, LTOT is prescribed for at least 15 h/day but has been recommended to be used continuously (24 h/day) based on a non-randomised comparison between the trial arms that suggested a relation between longer daily usage time and improved survival [3].

However, despite LTOT, patients with chronic respiratory failure have a poor prognosis, with a median survival time of about 1.9 years after starting the treatment [4], and also have a high burden of symptoms, adverse events and poor health-related quality of life [5].

The LTOT can be burdensome for many patients, as they have to be connected to the equipment or carry it with them. This burden and limitation could be particularly great for people prescribed to use it 24 h/day – their LTOT may become a ‘ball and chain’ [6]. Therefore, there was a great need of an RCT that directly compared the effect of LTOT prescribed for 24 or 15 h/day.

The REgistry-based randomised controlled trial of treatment Duration and mortality in long-term OXygen therapy (REDOX) was a multicentre, phase IV, non-superiority R-RCT of LTOT prescribed for 24 *versus* 15 h/day, that was conducted using the Swedish National Registry for Respiratory Failure (Swedevox) [7]. Published in the *New England Journal of Medicine* [8], REDOX was the first trial of LTOT in people with severe hypoxemia in over 40 years, and the first trial in this condition to include people with conditions other than COPD. Given the challenges in completing trials of oxygen therapy and in people with advance respiratory disease [9], experiences, challenges and solutions throughout the 10 years of conducting REDOX may be of value for researchers planning and conducting future R-RCTs within respiratory medicine.

The aim of this article is to describe the R-RCT methodology and our experiences throughout the 10-year period of planning and conducting REDOX, focusing on the challenges, their potential solutions and advice for planning future R-RCTs.

## The R-RCT methodology

### A paradigm shift in research

The interest in conducting R-RCTs has increased rapidly over the recent decades, as a pragmatic and feasible methodology to address some limitations of ordinary RCTs. Traditional RCTs in respiratory disease are reported to have a very low external validity with less than 5% represented by patients from clinical real life [9]. The R-RCT has emerged as a scientific paradigm shift, with the potential to accomplish large trials with adequate power at relatively low costs, with high generalisability, and more complete enrollment and follow-up than conventional RCTs [10,11]. In 2013, the Respiratory Effectiveness Group collaborators suggested a two-dimensional framework to classify clinical trials, where the R-RCT was regarded as the most appropriate form of clinical study as it includes both a pure, conformed study-population and a high external validity due to inclusion of the majority of the source population [12].

### Defining R-RCTs

The concept of the R-RCT was developed by Swedish cardiologists using national quality registers, where an R-RCT was defined specifically as a clinical trial where inclusion and randomisation is performed entirely within a registry at the consecutive registration of new patients [11]. An often-cited review has described an R-RCT as a pragmatic trial where a registry is used as a platform for case records, data collection, randomisation, and follow-up [10]. More recently, CONSORT-ROUTINE guidelines for RCTs using routinely collected data were published. In this report, the concept of R-RCTs was described more generally as trials embedded in registries [13]. Recent systematic reviews indicate that in reality, the term R-RCT is very heterogeneously used. In its widest form, it encompasses all clinical trials where a registry is partially used, including identification of eligible participants, active enrollment at entering into a registry or collection of baseline characteristics or outcome data [14,15].

### Pioneer R-RCTs

The use of Swedish cardiovascular quality registers has in a short period resulted in several high-quality trials. Examples include the TASTE study of percutaneous coronary intervention (PCI) with or without initial manual thrombus aspiration in patients with ST-elevation myocardial infarctions [16], the VALIDATE-SWEDEHEART study of bivalirudin versus heparin in patients, with myocardial infarction undergoing PCI [17], the DETO^2^X study of treatment with oxygen *versus* ambient air in acute myocardial infarctions without severe hypoxemia [18], several studies on technical procedures of PCI [19,20] and the TIMING study of early *versus* delayed intervention of non-vitamin K oral anticoagulants after cerebral infarctions in patients with atrial fibrillation [21].

Using the broader definition of an R-RCT (using registries for any of the trial processes), a much higher number of R-RCTs has been performed covering several specialties. The most common specialties so far are cardiology and oncology, and R-RCTs have most often been conducted in the USA and in the Scandinavian countries [15].

### Strengths and limitations of R-RCTs

The typical features, strengths and limitations of R-RCTs are summarised in Table 1. Ideally, an R-RCT identifies, randomises and collects both baseline and outcome data within a registry. The pragmatic design is suitable for comparison of two or more existing treatment alternatives, but not for early phase studies where increased attention and surveillance is needed. The obvious advantage of R-RCTs is the possibility to include all kinds of patients including old, frail and multi-morbid patients in daily clinical practice, at low cost in spite of high recruitment and completion of follow-up. Major disadvantages are that the quality of the study depends on the quality and coverage of the registry.

**Table 1.** Features, strengths and limitations of R-RCTs.

Features	Strengths	Limitations
Identification of participants using a registry Randomisation and electronic case report forms within a registry Collection of baseline data from a registry Collection of outcome data from a registry Typically performed to compare effectiveness of two treatment options	High external validity and generalisability Low costs Feasible for recruitment in clinical practice Rapid consecutive recruitment High completeness of follow-up	Non-mandatory registries may have low coverage Accuracy of reported baseline data may differ, Registries may have missing data Often need of completion with data from other sources like questionnaires Potential ethical issues like data safety and handling of consent and withdrawal

## REDOX

**Figure 1.**
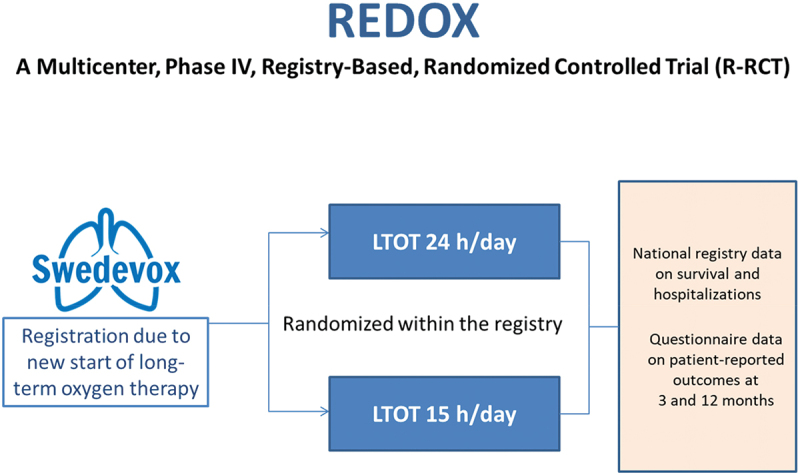
Final design of the REDOX trial.

The REDOX was designed as a pragmatic, non-superiority trial to evaluate whether LTOT prescribed for 24 h/day, as compared with LTOT 15 h/day, did not reduce the risk of all-cause hospitalisation or death up to 1 year [8]. Or, specifically, this was the way the trial was designed finally [8], after having been changed from its originally published design [7] to address challenges that emerged throughout the study period. The final trial outline is shown in Figure 1. REDOX was prospectively registered (Clinical Trials: NCT03441204). Challenges, solutions and advice related to each part of the trial conduct are described below and summarized in Table 2.

**Table 2.** Challenges and solutions in the REDOX trial.

Challenge	Solution
Need of filling an important knowledge gap	Pragmatic trial comparing two clinical alternatives
Management	UCR manager and team
Costs	Applications for grants, no final compensations to the sites, limited follow-up to one year
Electronic data capture	Electronic data capture system – data capture in separate database; use more pragmatic data capture solutions with post-trial cross-linkage
Recruitment	The changes, the drop in number; experienced a late rush; one dominating centre (the importance of investigator and staff effort and centre size)
Need for continuous amendments	Don’t give up!
Frail population and resistance to the research question and interventions	Research network with implementation of knowledge about what is known and what is not known Pre observational studies
Reporting adverse events would be time-consuming	Data on burn injuries, fall injuries, and nosebleeds from the National Patient Registry
No objective measurement on oxygen use	Information that self-reported use was anonymous and not forwarded to the responsible physician

### Selecting the trial aim

The R-RCT design is optimal to compare interventions that are feasible to apply and are treatment options in the current clinical practice (such as LTOT prescribed for 24 or 15 h/day [4]), where head-to-head comparisons of effects are needed. The population should be people routinely included in a health registry with high coverage and completeness of patients and variables. The primary outcome should optimally also be registry-based, with high coverage and high-quality data, such as the hard endpoint of all-cause hospitalisation and mortality [11]. The aims of REDOX were developed in discussion with national and international experts in the field.

### Design, approvals, and costs

**Figure 2.**
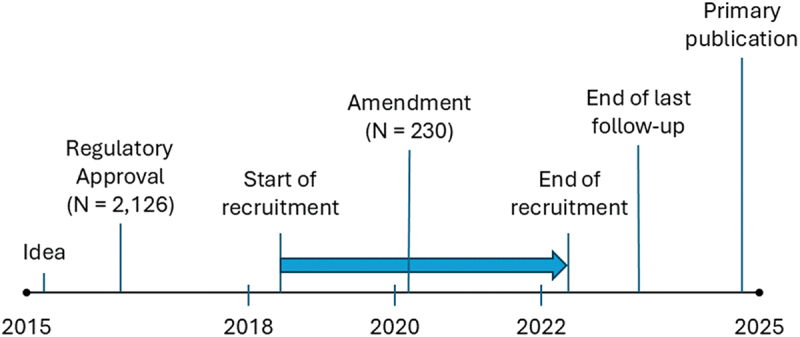
Time line for conducting the REDOX trial. The blue arrow depicts the time period of recruitment.

REDOX was planned and conducted over a ten-year period (Figure 2). The choice of trial design and intervention(s) to evaluate can have important implications on the regulatory requirements. At the time of designing REDOX in 2015–2016, Swedish regulatory authorities considered liquid oxygen and compressed gas cylinders, but not oxygen concentrators, as pharmacological interventions. As REDOX included patients starting LTOT using *any* of the delivery methods, it was required to be designed, registered, followed up and monitored in accordance with Good Clinical Practice for a pharmacological trial [22]. For comparison, the same extensive administrative processes were not required for a concurrently designed trial of ambulatory oxygen therapy using portable oxygen concentrators [23].

Preliminary data supporting the equipoise of trial interventions was of fundamental importance to obtain regulatory approval to conduct REDOX. Using the existing observational data in the registry, we could show that there were no apparent difference between patients in the current clinical practice who were prescribed LTOT 24 h/day or 15–16 h/day in terms of risk of hospitalisations [24] and mortality [4].

R-RCTs require substantial and sustained funding, but the administrative burden and costs for R-RCTs can be considerably lowered due to the use of an existing registry infrastructure for screening, enrollment, data collection and follow-up. In REDOX, the main costs were for administrative support, building and incorporating a module in the registry for running the trial, monitoring, database management, registry cross-linkage, and data quality control. Decisions at each design step can influence costs markedly, such as decisions of whether to embed the trial module into the registry (higher costs but may increase feasibility); the amount of non-registry data to be collected during the trial, and the trial duration. A way to decrease costs in future studies can be to keep the randomisation and data capture module separate from the registry (not embedded).

A factor that decreased costs in REDOX was that the intervention was free of cost for the patients (except electricity costs) and feasible to administer within clinical practice, while interventions involving expensive medications or techniques would need additional funding or industry collaborations.

### Interventions and assessments

In Sweden, patients starting LTOT are entered into the national registry Swedevox. As described above, a trial module was temporarily embedded during the REDOX study period. When entering a new patient, the responsible doctor was reminded of the trial, and if written informed consent was obtained, the patient was randomised immediately within the Swedevox registry in a 1:1 ratio, to one of the two treatment alternatives LTOT 24 h/day or 15 h/day (Figure 1). The allocated treatment group was unblinded for patients or staff but blinded by the statistician before lock of the database [8].

The primary outcome composite data were obtained from mandatory national registries like the National Patient Registry and Cause of Death Registry. Secondary outcome data on self-reported breathlessness, fatigue, health status, physical activity, cognitive status, actual oxygen use and preferred treatment option were obtained from postal questionnaires at 3 and 12 months [8].

A regulatory challenge in REDOX was that the Swedish Medical Products Agency required that any collection of data on adverse events had to be reported to regulatory authorities by the responsible units and not, as originally planned, using surveys. However, as both interventions were clinically established and their side effects well-known, adverse events known to be related to LTOT were accepted to be exempted from general reporting. Instead, we assessed diagnosed burn injuries, fall injuries, and nosebleeds post-hoc, at the time of publication, using the National Patient Registry [8]. This solution substantially impacted the burden and feasibility of the trial.

Since the Medical Product Agency required full monitoring as in a drug trial according to Good Clinical Practice throughout the study period, the trial duration was limited to 12 months after randomisation to limit costs. Assessments of longer-term outcomes, beyond 12 months, were not included in the trial protocol, as monitoring would be required for the longest period of follow-up.

All questionnaires were posted and managed by a central unit, Uppsala Clinical Research Centre). The main advantage of this distinction was to keep the study procedure at the study sites as simple as possible to be able to fulfill in daily clinical practice. Another important feature was that the patients could be informed that their individual responses could not be accessed by their doctor or oxygen nurse. This reduces the risk of information bias, as patients were expected to report true adherence to their allocated arm and not reports that they knew doctors and nurses would like to hear.

### Recruitment

The registry-based methodology provides advantages by facilitating patient identification, screening, and recruitment, even in frail patient groups, such as in people with chronic respiratory failure.

The number of patients starting LTOT in Swedevox has been stable around 1,000 patients per year in recent years. All units prescribing LTOT are connected to the Swedevox registry, which has an overall coverage of about 85% of all patients starting LTOT nationwide [25]. Patients fulfilling the established LTOT criteria for chronic severe hypoxemia [26,27] were eligible. Exclusion criteria included active smoking, anticipated repeated contact with fire, inability to safely comply or lack of Swedish social security numbers, or opting out from being registered in Swedevox.

Based on the original power calculation, our intention was to include 2,126 patients within 27 months [7]. However, the initial recruitment rate was much lower than expected, with only 5% of the final study population included after 2 years. An expected reason for insufficient recruitment was that some patients were denied participation because they preferred a specific treatment duration and did not want to be randomised with the risk of being allocated to the other arm. However, the major reasons for the screening failure were external, including lack of staff or that the local investigator was not available. Another reason was that the investigator judged that the patient could not be randomised for medical reasons, which may reflect prevailing perceptions about the potential efficacy and safety of oxygen therapy and treatment traditions. Due to the slow recruitment, the trial protocol was amended.

### Amendments to the trial protocol

Due to the slow inclusion rate, the statistical analysis plan and sample size were revised during the study period. This var justified as conduct if the trial itself was entirely unaltered.

After 110 patients were included, an amendment was submitted to the Medical Product Agency, which was approved 10^th^ of February 2020 (5.1–2020–6277). In the updated protocol, the primary outcome was changed from all-cause mortality to a composite of all-cause hospitalisation or death; the primary analysis population was changed from patients with COPD to all randomised patients; and the limit for non-superiority was defined as the lower 90% confidence interval of the hazard ratio being >0.67 instead of >0.83 [8]. These changes resulted in a new planned sample size to 230 randomised patients. All amendments pertained to the statistical analyses only. No changes were made to eligibility criteria, data collection or to trial conduct.

### Conduct of the trial

The conduct and completion of the REDOX trial were dependent on the services provided by the central unit, Uppsala Clinical Research Centre, including administrative support; trial management; development, testing, implementation and maintenance of the trial module in the Swedevox registry; management of postal patient surveys; database management and quality control; trial monitoring; registry data cross-linkage and statistical analyses.

**Figure 3.**
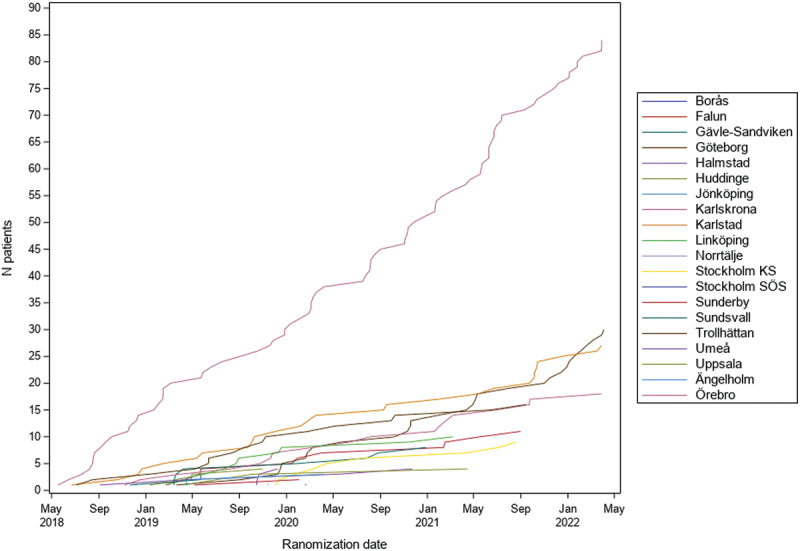
Recruitment over time by study centre in the REDOX trial.

As REDOX was conducted within routine clinical care, recruitment differed markedly between participating centres (Figure 3) owing mainly to availability over time in staff, resources, and involvement and time of the Investigators and oxygen nurses to inform and enroll patients.

The challenges in recruitment also reflect known difficulties in conducting trials of oxygen therapy [9], where both health professionals, patients and caregivers often had strong convictions regarding the potential efficacy of oxygen therapy and of the safety of withholding oxygen therapy up to 9 hours during daytime in patients with chronic severe hypoxemia. Additionally, many patients wanted to determine when to use their oxygen therapy and said that they would not be willing to be randomised between 24 and 15 h/day, and therefore could not be included in the trial. Similar challenges regarding patients’ oxygen preferences were reported in the LOTT [9]. Important factors to complete REDOX despite these challenges were: 1) careful information to the participants and Investigators of the equipoise of the trial interventions – and for this, the published observational data of similar outcomes in people prescribed the different daily oxygen durations in the registry [4,24] were crucial; 2) optimising statistical analyses to reduce the sample size needed as much as possible; and 3) the registry-based design that made recruitment more feasible and less costly – enabling recruitment over a longer time period to complete the trial.

A concern in the Investigator group was that challenges in recruitment could lead to selection bias, for example, where patients with more severe hypoxemia would be less likely to be enrolled. A strength of the R-RCT design is that the characteristics and registry-based outcomes can be compared between the included trial population and all patients starting the therapy (LTOT) in the registry during the study period. This comparison showed that characteristics were very similar, arguing against significant selection bias and supporting the idea that the findings have high external validity.

### Analysis and reporting

A final database for analysis was created by merging the data from the trial module, data from the postal questionnaires and cross-linkage with national mandatory registry data for the follow-up period. A statistical analysis plan was defined in collaboration with the trial biostatistician and published on ClinicalTrials.gov before the analysis database was available. The statistical analysis plan and the first manuscript version included analyses of the primary and main secondary registry-based outcomes. However, *the New England Journal of Medicine* required that analyses of other secondary outcomes, including of patient-reported questionnaire data, to be included in the primary efficacy article [8].

Planned further articles include a more detailed analysis of the risk of incidental diseases between the treatment groups as well as of the socioeconomic impact of the findings. A long-term follow-up using registry data is planned to be conducted (preliminary 5 years after randomisation).

### Summary and looking ahead

REDOX is the first trial of LTOT in severe hypoxemia since the 1970s and could be completed largely owing to the registry-based design. R-RCTs can facilitate trials and improved evidence also in frail populations with very severe and end-stage disease. However, R-RCTs come with advantages as well as challenges. This article has showcased our challenges and the solutions to try to combat and overcome these challenges throughout REDOX. Building on these experiences, we are now conducting another R-RCT evaluating the effect of oxygen therapy with high-flow equipment during nighttime in people with LTOT (ClinicalTrials.gov: NCT06247397). To decrease administrative burden and costs in this trial, participants are identified and screened using the national Swedevox registry, but the trial module is kept separate from the registry, and all data transfer from and cross-linkage with registry data will be performed after recruitment closure. We hope the information and discussions in this article will be useful for investigators designing new R-RCT endeavors.
